# Eye-Hand Coordination Patterns of Intermediate and Novice Surgeons in a Simulation-Based Endoscopic Surgery Training Environment

**DOI:** 10.16910/jemr.11.6.1

**Published:** 2018-11-08

**Authors:** Damla Topalli, Nergiz Ercil Cagiltay

**Affiliations:** Atilim University, Turkey

**Keywords:** Eye movement, eye tracking, saccades, gaze, hand-movement, haptic device, eye-hand coordination, surgical skill assessment

## Abstract

Endoscopic surgery procedures require specific skills, such as eye-hand coordination to be developed. Current education programs are facing with problems to provide appropriate skill improvement and assessment methods in this field. This study aims to propose objec-tive metrics for hand-movement skills and assess eye-hand coordination. An experimental study is conducted with 15 surgical residents to test the newly proposed measures. Two computer-based both-handed endoscopic surgery practice scenarios are developed in a simulation environment to gather the participants’ eye-gaze data with the help of an eye tracker as well as the related hand movement data through haptic interfaces. Additionally, participants’ eye-hand coordination skills are analyzed. The results indicate higher correla-tions in the intermediates’ eye-hand movements compared to the novices. An increase in intermediates’ visual concentration leads to smoother hand movements. Similarly, the novices’ hand movements are shown to remain at a standstill. After the first round of practice, all participants’ eye-hand coordination skills are improved on the specific task targeted in this study. According to these results, it can be concluded that the proposed metrics can potentially provide some additional insights about trainees’ eye-hand coordi-nation skills and help instructional system designers to better address training requirements.

## Introduction

Minimal invasive surgery (MIS) is preferred by surgeons and patients as it has several benefits for the patients, causing less infection risk and shorter operation time (1). However, MIS education for surgeon faces many challenges, such as difficulty to learn, while more than 30 procedures for the learning curves have been reported (2, 3). In this respect, depending on the type of the operation, the number of such procedures can be increased up to 100 (4).

Surgeons involved in these types of operations need to develop specific psychomotor skills, such as eye-hand coordination, and depth perception to mention a few (5, 6). As the location of the operation site can only be observed through a monitor in MIS, mislocation (e.g., in case that the display in the monitor through the endoscope and hands controlling the operational tool are at different locations) can make it impossible for the surgeon to follow his/her hands as well as the operative scene simultaneously (7, 8 ). This mislocation problem can be more critical in complex surgery procedures, such that surgeons might even require assistance to control the endoscope.

Hence, surgical education programs today require very important knowledge and skills to be gained. In traditional training programs, these skills are gained in the operating theatre and upon practice on patients. According to earlier studies, critical errors (9) can occur when these skills are not developed properly or urgently put in practice (10). In current education programs, novice surgeons should improve their skills and practical abilities on real patients supervised by experts (11), which is an expensive and very risky method. Apart from these factors, assessment of surgical skills is carried out by training specialists through structured schemes, such as OSATS (Objective structured assessment of technical skill), which is a method for testing specific operative skills in surgical trainees (12, 13 ). However, as also reported by Moorthy et al. (14) there are certain constraints in this method, such as resources and time to find supervising surgeons to observe and evaluate the performance of trainees (14). Naturally, since these operations directly affect human safety, ethical issues may arise. As a result, in order to guarantee patient safety, trainees should be educated by other means than with actual patients.

In addition, the use of movement based measures has been studied extensively, and has been suggested as an effective method for monitoring surgery training (15). It has been reported that motion analysis devices are useful tools to assess performance compared to merely relying on OSATS and time (6). Tracking hand and instrument movements using markers, known as ‘motion analysis’, has been suggested by earlier studies as an alternative method to OSATS in assessing the related skills by measuring the economy of movement (16). Latko et al. (1997) studied videotaped and documented hand activities rated from 0 to 10 through observations, and provided some definitions for hand movements. According to them, when no regular exertions are detected, the hand activity is considered as ‘idle’, and when there is infrequent motion, it is considered as ‘steady motion’. Based on the frequency of the motion, they also propose ‘consistent conspicuous’ (long pauses or slow motions), ‘slow steady motion’, and ‘rapid steady motion’ (17).

Besides, several hand-movement metrics have been proposed, such as path length, motion smoothness, depth perception (as the total distance traveled by the instrument along the instruments’ axis), response orientation and grasping (15). In this vein, some studies have been carried out on motor behaviors in surgical skills based mainly on the path-length, the amount of time to complete a procedure, and idle time, which topic needs to be considered but has remained rather neglected (18). Oropesa et al. (2011) define ‘idle time’ as lack of movement in both hands representing the delay in motor planning or decision making (19). One example of motion metrics is the smoothness of hand function. Oropesa et al. (2013) define ‘motion’ as abrupt changes in acceleration resulting in jerky movements of the instrument (19, 20 ). Another proposed metric, ‘working space’, is suggested for the economy of the area and economy of volume efficiency in MIS (20), and is determined by using an electromagnetic sensor to track the participants’ hand movements and the summation of distances from the sensor’s average spatial location (21). However, further research has been reported to be necessary in a recent study to better understand the role and usage of psychomotor metrics, such as smoothness, to assess performance during certain medical procedures (21).

Detecting the location of a given object in a precise manner is, such as a tool or hand, important for each of these metrics (20, 22 ). In the literature, video-processing methods (23, 24 ) and motion-tracking systems (19) have been proposed to detect the position of the tool in a precise way, giving rise to other practical concerns.

Despite the presence of evidence showing a correlation between motor skills and eye events (25, 26, 27, 28, 29), there are very limited studies conducted to improve the understanding and objective measuring of surgical residents’ eye-hand coordination skills, while there is hardly any standardized measure in this regard, thus limiting the interpretation and generalization of related results (30). Hence, despite the fact that there exist some metrics providing insights into the relationship between the eye and the hand as well as their coordination, still there is a need to extend our knowledge of how hand movements are guided and controlled by vision (31). Objective methods for analyzing surgeons’ hand movement patterns have not thoroughly led to devising fully satisfactory methods of assessment (32); whereas such metrics are necessary in order to provide proper feedback and continuous analysis of psychomotor skills in MIS (12, 33 ).

Today, computer-based simulation environments offer many benefits since they are cheaper, provide more time for practice and can be easily modified for different rare cases (34). Apart from such environments, in the literature, there are several studies showing that eye-tracking technology provides beneficial insights for surgical training purposes. In a review study, Tien et al. (2014) concluded that eye tracking offers reliable quantitative data for objective assessment purposes to improve performance in surgical training (35). Several studies have been conducted to better understand the eye-movement behaviors of surgical residents for improved skill assessment purposes. Research suggests that recording these eye movements may be beneficial both for skill assessment and training in the field of surgery (5). Today, computer-based simulation environments provide several objective assessment capabilities, which can be attained continuously during the training period and without expert supervision (36). However, the calculation methods for many of these metrics are implicit and within the scope of commercial simulators, making them difficult to manipulate.

In light of all these shortcomings and needs within the field of surgery, the present study first attempts to adapt the eye-movement event analysis approaches to the hand motion and propose new additional objective metrics for the hand-movement events. Additionally, through haptic devices the data pertaining to the hand locations of the surgical residents are collected along with their eye-movements while performing surgical tasks in a virtual reality environment. By analyzing both the eye and hand behaviors of the surgical residents, this study aims to understand the eye-hand coordination skills of the surgical residents in a more elaborate way.

## Methods

The aim of this study is to assess the relationship between eye-gaze and hand-motion metrics to understand the eye-hand coordination behavior differences of intermediate and novice surgeons in a simulation-based endoscopic surgery environment.

The findings of the literature imply that there are several different algorithms which use constant threshold values to classify eye events into fixations and saccades. However, it is also reported that an ideal algorithm should automatically identify the threshold values, without requiring any parameter setting from the user (37). For instance, the BIT (Binocular-Individual Threshold) algorithm is a fully automatic, velocity-based algorithm to determine fixations (i.e., fixation duration and fixation number) and saccades from the eye data, using task- and individual-specific thresholds (38). The algorithm is regarded as ‘machine and sampling frequency independent’ (38).

Due to the ability of automatically defining task-specific thresholds, this algorithm is more suitable for skill-based studies. Hence, in this study, the BIT algorithm is used to identify the fixation duration, fixation number and the saccades of the eye-gaze data. BIT is also used to classify hand movement events using the data collected within a surgical simulation environment. Since the eye and hand can move at different velocities, the threshold values should be determined automatically for each case with this algorithm. The source code of the algorithm is available on the authors’ website, using MATLAB (38).

### Participants

A total of 15 surgical residents (ten surgeons and five interns) from the Department of Neurosurgery (six participants) and Otolaryngology (ENT) (four participants) from Hacettepe Medical School in Ankara, Turkey participated in this study. There were two skill level groups of participants, intermediate and novice, based on the categorization presented in Silvennoinen et al.’s study (39). In that concern, those who have operated at least one endoscopic surgery are considered as ‘intermediate’, whereas others who have only observed and assisted in endoscopic operations, but have not performed any surgeries by themselves, are considered as ‘novice’. As shown in Table 1, most of the participants were male (86.66%).

**Table 1.** Information about Participants

**Skill Level**	**Age**	**Department**		**Gender**	
		**NRS**	**ENT**	**F**	**M**
Intermediate	28.4	1	5	1	4
Novice	25.6	4	5	1	9
NRS: Neurosurgery					
ENT: Ear Nose Throat					

Detailed information about these participants according to their endoscopic surgical expertise (average number of operations observed, assisted and performed) is given in Table 2.

**Table 2.** Participants’ Surgical Experience

**Skill Level**	**Average number of Endoscopic Surgery**
	**Observed**	**Assisted**	**Performed**
Intermediate	52.0	39.6	23.8
Novice	8.2	1.0	0.0

### Apparatus

**Figure. 1:**
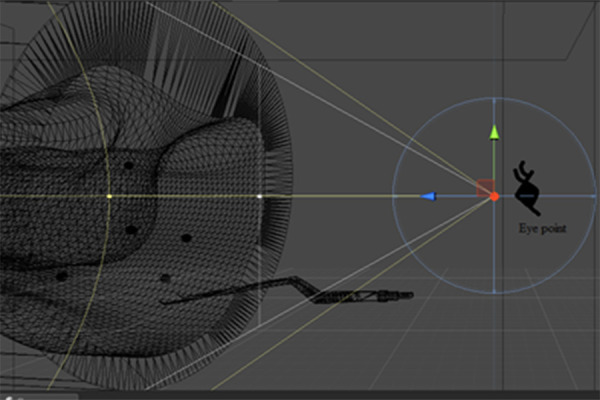
Camera’s FOV in Simulation Environment

Haptic devices can be integrated into training simulations for MIS procedures (40) in order to provide similar and real-world practice as well as a realistic sense of touch. Accordingly, in this study, a mid-range professional ‘Geomagic Touch’ haptic device is used to perform the tasks in the scenarios. The simulation software recorded a hundred data points per second as the hand coordinates for both hands with the help of these haptic devices. Additionally, the eye movement data is gathered by using a 60 Hz eye-tracking device, the Eye Tribe (41), which is easy to set up, transportable and reported as appropriate for use in scientific research (42). This tool is used to track the user’s eye movements and calculate the on-screen gaze coordinates with an accuracy of 0.5°-0.7°. During the experiment, eye-gaze coordinates were gathered in a top-left oriented 2D coordinate system. The screen resolution is 1920 x 1080 pixels; in other words, the horizontal field of view (x-coordinate) is 1920 pixels, whereas the vertical field of view (y-coordinate) is 1080 pixels. The field of view (FOV) of the camera, from the left-perspective for Scenario-2 can be seen in Figure 1.

**Figure. 2:**
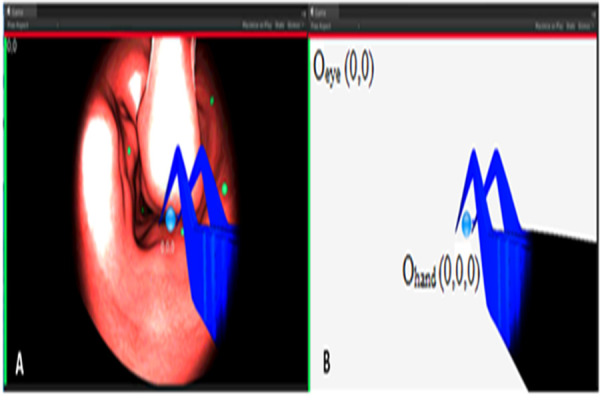
Eye-Gaze and Hand Motion Coordinates in Scenario-2

In our software, hand motion coordinates, regarded as the position of tool and camera in the scenarios, were represented as 3D vectors. Accordingly, the origins for eye O_eye_ and for hand O_hand_ coordinates have been represented in a 2D scene and appear in Figure 2-A and B.

Due to the low sampling rate, the analysis of saccades may become problematic when collecting data using the Eye Tribe tracker. It is reported that measuring saccade metrics require high frequency sampling (43). However, it has also been stated that velocity-based algorithms for saccade detection that works on high-frequency data do a relatively good job on the Eye Tribe data (43, 44 ), but still they fail to be accurate when it comes to saccadic positions (43). However, there are a number of studies in the literature using 50 Hz (38) and 60 Hz sampling rate trackers in order to classify eye events into fixations and saccades (44).

### Design

**Figure. 3:**
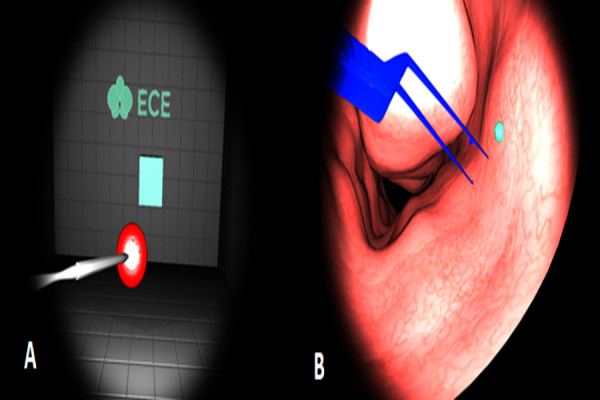
Scenarios for Experimental Study

In the experimental study, there were two both-handed scenarios for the surgical training process used. The first one was prepared to practice on general skills, such as learning the use of surgical tools with the endoscope and developing depth-perception in a simulated 3D environment (Scenario-1). The other scenario closer to the operational procedures uses a simulated anatomical nose model (Scenario-2). Each scenario consists of ten repetitive tasks. The layout of the scenarios is shown in Figure 3-A and B, respectively.

A: Scenario-1: Moving the Red Ball into the Box

B: Scenario-2: Clearing the Nose

### ‘Moving the Red Ball into the Box’ Scenario

In this scenario (Scenario-1), each participant is asked to approach the red ball with the haptic device, catch it, and then move it into the green box as shown in Figure 3-A. The tool is controlled with the dominant hand of the participant, whereas the camera is controlled by his/her non-dominant hand. The position of the ball and the box changes arbitrarily in each trial. The participant must complete this process successfully, which includes 10 tasks, within the allocated time period. In case of failure to complete each task within 10 seconds, the ball and the box disappear.

### ‘Clearing the Nose’ Scenario

In this scenario (Scenario-2), the participant must remove the green ball-like objects, which are spread through the nose model as shown in Figure 3-B. The camera is used as the light source and the cautery model as the tool to collect the objects. In case of a collision - that is, if the haptic device touches the tissue - it provides a force feedback that feels as if the device pushes back in the hands of the user.

This study is conducted as part of a research project and the content of both scenarios are prepared based on opinions of the neurosurgery and ENT domain experts. Mainly endoscopic pituitary surgery procedures are aimed to be practiced which is in the scope of both domains and performed starting from the nose holes through the pituitary area. Accordingly, the scenarios are designed for beginners of these operations.

### Procedure

**Figure. 4:**
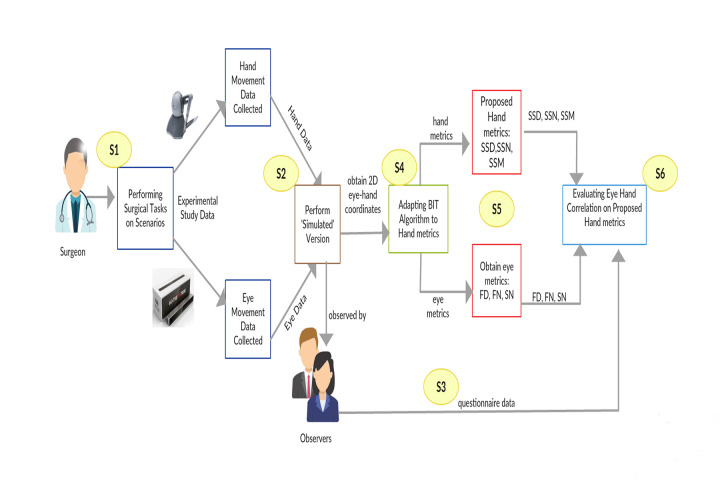
Procedure of the Experimental Study

As shown in Figure 4, in this study, the research procedure mainly consisted of five stages. These are S1: Experimental study; S2: regenerated simulation version; S3: BIT algorithm; S4: Eye and hand metrics; and S5: Eye-hand coordination analyses.

**Figure. 5:**
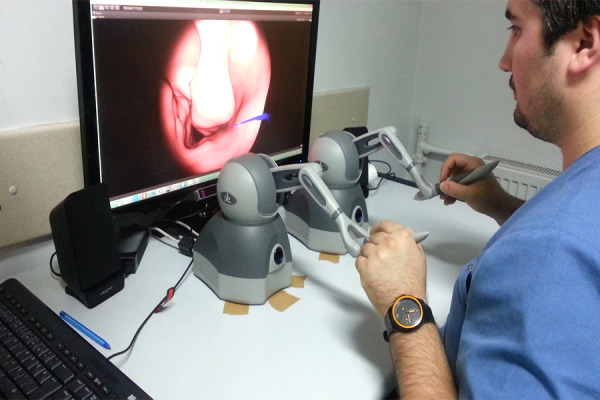
Experimental Setup

At the beginning of the experiment, the participants were asked to fill out a questionnaire including their demographic information, dominant-hand and experience level (i.e., years in the department, and the number of operations observed, assisted, and performed). Prior to the experimental study, the participant is seated 70 cm. away from the screen. First, each participant was informed about the calibration process; more specifically that they should maintain the distance and that they are not allowed to move their heads or body once calibration is completed. After this briefing, the calibration process started. Nine calibration points appeared on the screen one after the other, with a viewing time of two seconds each. At the end, a five-star rating is displayed to provide the accuracy of the calibration. If the result of the calibration is four-star (<0.7°) or five-star (<0.5°), then it is regarded as acceptable for the experimental study. After that, a brief instructional video explaining how to perform the task was shown to each participant separately about the procedure. Next, each participant was asked to perform two scenarios, Scenario 1 and 2, using both their dominant and non-dominant hands at the same time. The eye-gaze data (i.e., pupil size, fixation, raw and smoothed X and Y coordinates of both left and right eye) and hand motion data (i.e., tool and camera position, tool and camera rotation as 3D vectors) were collected and stored using a special software (Figure 4: S1). The experimental setup can be seen in Figure 5.

**Figure. 6:**
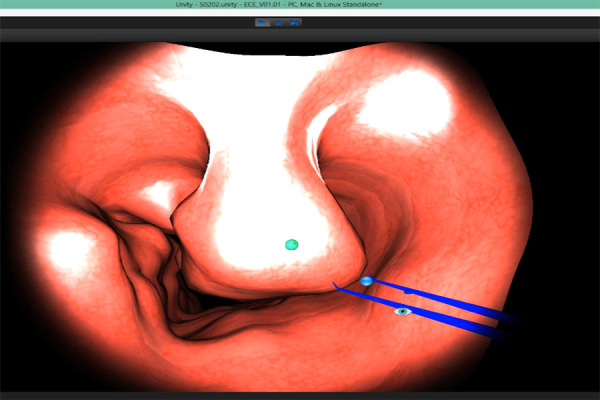
‘Simulated’ Performance of a Participant (Scenario-2)

Afterwards, the performance of each participant in both scenarios is regenerated in the simulation environment using a software developed in the Unity environment (Figure 4: S2). This regeneration software took each participant’s eye data with the help of the tracker device and the hand data from the haptic device while they were performing each task during the experimental study. In this way, each participant’s eye and hand coordinates are aligned within the same time scale by representing the eye location through an eye icon and hand location through a small blue sphere in the regenerated simulation re-play (See Figure 6).

This regenerated simulation allows researchers to determine the extent regarding tissue-contact, left-right hand coordination and the eye-hand coordination of each individual. In the meantime, the observation data was gathered through a questionnaire to understand such behavior differences between novice and intermediate groups. Five observers (other than the researchers) have evaluated the participants’ performances in Scenario-2. As this scenario is based on an environment similar to the operational procedures (Figure 4: S3), the observations are conducted on this scenario. All of the observers are the graduate students in the field of engineering and briefed about the observation procedure before they start the evaluations.

Using this approach, the raw coordinates for both the eye and hand movement data aligned in the time scale were obtained as the output for regenerated simulated version of the scenarios. Then, these coordinates were given as an input to the BIT Algorithm (Figure 4: S4). Afterwards, the classification process of the eye and hand movements - as either fixation or saccade events- were identified by running the algorithm, separately for the eye data and the hand data. The output of the BIT algorithm performed on the eye data is the fixation and saccade metrics, whereas metrics related hand movement were obtained as the outputs from the hand data (Figure 4: S5). Finally, all collected data is analyzed to better understand the efficiency of the proposed metrics and to objectively measure the eye-hand coordination skills of the participants (Figure 4: S6).

### Metrics

In this study, using the BIT algorithm, three metrics are identified, namely Fixation Duration (FD) (the time from one saccade to another), Fixation Number (FN) (the number of fixations in an interval) and Saccade Number (SN) (the number of saccades in an interval).

As explained in the procedure, the eye movements and the hand movements of the participants are aligned in the same time scale. The authors believe that further insight regarding the eye-movement events can be also be applied to the hand movement events as well. Our main aim in this study is to test this assumption by comparing the results conducted in this study with the ones that are reported in earlier studies on eye-hand coordination skills of the surgeons. Accordingly, in the present work, new hand metrics are introduced to identify the hand movements of the participants as explained below:

The ‘Stand Still’ metric is proposed in this study as the period when the hand movement remains within a very small range and lower velocity for some time. In other words, it determines the ‘idle state’ of the hand movement. By running the BIT algorithm, such events can be classified into ‘Stand Still Duration’ (SSD) and ‘Stand Still Number’ (SSN) for hand movements. In this respect, the ‘Sudden Sharp Movement’ (SSM) metric is also proposed to identify very fast, sharp hand movements while performing any given task.

## Results

A correlation analysis is performed to assess the relationship between eye-gaze and hand-motion metrics considering three pairs: the fixation duration of the eye-gaze (FD) and stand still duration of the hand motion (SSD), the fixation number of the eye-gaze (FN) and stand still number of the hand motion (SSN), and the saccade number of the eye-gaze (SN) and the sudden sharp movement (SSM) for hand motion, where the correlation coefficient r is calculated. The value of | r | from 0.1 to 0.3 represents a small correlation. From 0.3 to 0.5, the value represents a moderate correlation, while larger than 0.5 shows a strong correlation as reported by Cohen (45, 46 ).

### Eye- Hand Correlation Results for Scenario-1

**Figure. 7:**
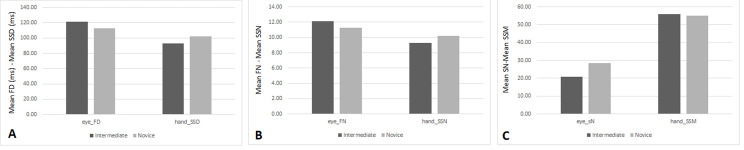
Mean for Eye and Hand Metrics for each group in Scenario-1

In Scenario-1, the descriptive statistics for the two groups of participants (intermediate and novice) for both the eye metrics (FD, FN, and SN), and the hand metrics (SSD, SSM and SSN) are depicted in Figure 7. Additionally, the mean and standard deviations for all metrics are in Scenario-1 is shown in Table 3.

A: Scenario-1: Mean FD (ms) of eye and Mean SSD (ms) of hand

B: Scenario-1: Mean FN of eye and Mean SSN of hand

C: Scenario-1: Mean SN of eye and Mean SSM of hand 

**Table 3.** Descriptive Results for the Metrics in Scenario-1

	**Intermediate**		**Novice**			
***Eye Metrics***	**M**	**SD**	**M**	**SD**
FD	121.53	19.05	112.70	26.55
FN	12.14	1.91	11.26	2.66
SN	20.80	13.46	28.60	32.46
***Hand Metrics***	**M**	**SD**	**M**	**SD**
SSD	92.99	10.25	101.98	15.03
SSN	9.30	1.01	10.19	1.51
SSM	55.80	37.48	55.10	15.82

### Eye-Hand Correlation for Intermediates

A Pearson's product-moment correlation was run to assess the relationship between the eye-gaze and hand motion metrics, (FD- SSD and FN- SSN) and saccades (SN- SSM) for intermediates. For all of these three pairs, the preliminary analyses showed the relationship to be linear with both variables normally distributed, as assessed by the Shapiro-Wilk's test (p > .05), and there were no outliers. Also, there was a strong negative correlation between the FD – SSD and FN- SSN metrics among the intermediates, r = -.836 and r = -.837, respectively. On the other hand, a strong positive correlation existed between the saccade metrics SN and SSM in the same group r = .755 (Table 4).

### Eye-Hand Correlation for Novices

Again, a Pearson's product-moment correlation was run to assess the relationship between the eye-gaze and hand motion metrics, and fixations (FD- SSD and FN- SSN) and saccades (SN- SSM) for novices. For the first two pairs related to fixation, preliminary analyses showed the relationship to be linear with both variables normally distributed, as assessed by the Shapiro-Wilk's test (p > .05) with no outliers. There was a moderate positive correlation for both the FD- SSD and FN- SSN metrics among the novice participants, r = .448. However, not all variables were normally distributed for the saccade metrics SN and SSM, as assessed by the Shapiro-Wilk's test (p < .05). Accordingly, a Spearman's rank-order correlation was run to assess the relationship between the saccade number of eye-gaze data and the sudden sharp movements of the hand data, with results showing a strong positive correlation for the SN and SSM measures, rs = .590 (Table 4).

**Table 4.** Eye- Hand Correlation Results for Scenario-1

**Skill Level**	**FD - SSD**	**FN - SSN**	**SN - SSM**
Intermediate	-.836 Strong-	-.837 Strong-	.755 Strong+
Novice	.448 Moderate+	.448 Moderate+	.590 Strong+

### Eye- Hand Correlation Results for Scenario-2

**Figure. 8:**
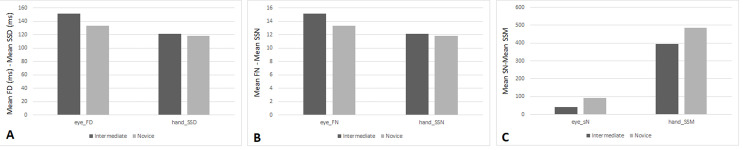
Mean for Eye and Hand Metrics for each group in Scenario-2

Figure 8 show the descriptive statistics for the eye (FD, FN, and SN) and hand metrics (SSD, SSM, SSN) for the two groups in Scenario-2. Additionally, the mean and standard deviations for all metrics are in Scenario-2 is depicted in Table 5.

A: Scenario-2: Mean FD (ms) of eye and Mean SSD (ms) of hand

B: Scenario-2: Mean FN of eye and Mean SSN of hand

C: Scenario-2: Mean SN of eye and Mean SSM of hand

**Table 5.** Descriptive Results for the Metrics in Scenario-2

	**Intermediate**		**Novice**	
***Eye Metrics***	**M**	**SD**	**M**	**SD**
FD	151.25	30.48	133.42	42.49
FN	15.11	3.04	13.34	4.24
SN	41.40	23.58	91.90	82.30
***Hand Metrics***	**M**	**SD**	**M**	**SD**
SSD	121.37	16.60	118.28	15.13
SSN	12.15	1.65	11.84	1.52
SSM	395.00	143.63	486.00	143.86

### Eye-Hand Correlation for Intermediates

A Spearman's rank-order correlation was run to assess the relationship between the FD- SSD and FN- SSN measures since not all the variables were normally distributed, as assessed by the Shapiro-Wilk's test (p < .05). There was a strong negative correlation for the pairs related to fixation that is the FD- SSD and FN- SSN metrics. In other words, an increase in the eye fixation duration and fixation number was strongly correlated with a decrease in the hand stand still duration and stand still number among the intermediates, rs = -.900, p < .05 (Table 6). However, both variables of the saccade metrics, SN and SSM, were normally distributed, as assessed by the Shapiro-Wilk's test (p > .05). Also, a Pearson’s correlation was run to assess the relationship between the saccade number of eye-gaze data and the sudden sharp movements of the hand data, with the outcome that there was also a strong positive correlation for the SN and SSM metrics, r = .846 (Table 6).

### Eye-Hand Correlation for Novices

A Pearson's product-moment correlation was run to assess the relationship between the eye-gaze and hand motion metrics, the fixation (FD- SSD and FN- SSN) and saccades (SN- SSM) for novices. For the first two pairs related to fixation, preliminary analyses showed the relationship to be linear with both variables normally distributed, as assessed by the Shapiro-Wilk's test (p > .05), and there were no outliers. There was a moderate negative correlation for both FD- SSD and FN- SSN metrics in novices, r = -.443 and -.441, respectively. However, not all variables were normally distributed for the saccade metrics SN and SSM, as assessed by the same test (p < .05). Accordingly, a Spearman's rank-order correlation was run to assess the relationship between the saccade number of the eye-gaze data and sudden sharp movements of the hand data. The results show a small positive correlation for the SN and SSM metrics for novices, rs = .06 (Table 6).

**Table 6.** Eye- Hand Correlation Results for Scenario-2

**Skill Level**	**FD - SSD**	**FN – SSN**	**SN - SSM**
Intermediate	-.900* Strong-	-.900* Strong-	.846 Strong+
Novice	-.443 Moderate-	-.441 Moderate-	.06 Small+

*Note that the correlation is significant at the 0.05 level

### Analyzing the Questionnaire Data

Five observers have monitored the participants’ performances in Scenario-2 in order to assess the left-right hand coordination and eye-hand coordination skills of the participants. Their evaluation is based on the items given in Table 7 to be ranked in accordance to five alternatives (1: Strongly Disagree, 5: Strongly agree) in a Likert scale-type questionnaire. 11 out of 15 participants (3 intermediates, 8 novices) were evaluated using the questionnaire data. The descriptive results appear in Table 7.

**Table 7.** Descriptive Results for Questionnaire Analysis of the Observers

	**Intermediate**		**Novice**	
**Questionnaire Item**	**M**	**SD**	**M**	**SD**
The participant shows developed depth perception skills in a 3D environment.	3.33	.42	2.12	.34
The participant shows developed skills in eye-hand coordination.	3.53	.30	1.92	.52

A Mann-Whitney U test was run to determine if there were any differences in the scores for the given expressions (Table 7) between the intermediate and novice groups. The distributions of the scores for intermediates and novices were found to be dissimilar among the participants once inspected visually. According to the results, considering these expressions, the 3D depth perception and eye-hand coordination skills of the intermediates (mean rank = 10.00) were significantly higher than novices (mean rank = 4.50), U=0, z= -2.461, p=.012.

## Discussion

The present study involved two main goals: first, to propose new objective metrics by adapting our knowledge in the field of eye-movement events; and, second, to test the appropriateness of these metrics in the endoscopic surgery field to objectively measure the eye-hand coordination skill levels of the surgeons.

Previous studies have attempted to evaluate the level of surgeons’ eye-hand coordination in the field of endoscopic surgery through some related task analysis (47, 48, 49) through eye movement analysis (31) or eye movements and arm movements (50). Hence, there are no current studies designed to understand the eye-hand coordination skills by analyzing both the hand movements and eye-movements of surgeons working in coordination. Accordingly, the proposed measures provide alternatives to understand such eye-hand coordination skills by analyzing the hand-movements and eye-movements of surgeons.

Based on the results of this study, in both scenarios, there exists a correlation between the average measured values of the three eye-gaze and hand motion metrics. The fixation metrics (FD-SSD and FN-SSN) are found strongly correlated for the intermediates, and moderately correlated for the novices. This outcome indicates that the novices require improvements in the related eye-hand skills. In other words, the intermediate participants’ eye-hand coordination skills are better improved compared to the novices. The results also show that, in Scenario-1, once the average fixation duration (FD) and the fixation number (FN) of intermediates increase, their average stand still duration (SSD) and stand still number (SSN) decreases. The increase in the fixation duration can be observed because of an increase in their concentration while performing the designated tasks. In other words, when their concentration increases, their hand movements become smoother and fewer occurrences of stand-still take place, indicating serial and smoother hand movements. This result supports the earlier studies which reported that skilled surgeons’ hand performances are more stable than less-experienced ones (51), and that the fixation number of experts are higher in task-relevant areas (52), but lower in task-redundant areas (52).

Different from intermediates, in both Scenario-1, an increase in the average fixation duration (FD) and fixation number (FN) of the eye-gaze among the novice participants correlates with an increase in their hand motion metrics (stand still duration (SSD) and stand still number (SSN)). At this stage, it can be inferred that the novices’ hand movements experience more idle or stand still states when their eye fixation increases. This finding is supportive of earlier results reporting that, while manipulating the tool, experts look directly at the target location, whereas novices track the movement of the tool until it reaches the target location (53). On the other hand, in Scenario-2, novices performed better in terms of eye-hand correlation, where the strength of the correlation remains the same as moderate, but the direction changes from positive to negative. This result indicates that in Scenario-2, when the eye fixation increases their hand movements also become smoother. This may be an indicator that their eye-hand movement coordination is improved for this specific task in Scenario-2 after practicing in Scenario -1.

A strong correlation occurs between SN and SSM in both scenarios for intermediates. In Scenario-2 the correlation coefficient increases slightly for this group. The result shows that, when there is an increase in the average number of saccades in the intermediates’ eye movements, their average sudden sharp movement also increases. Similarly, in Scenario-1 a strong correlation is also found for the novices on SN and SSM. However, in Scenario-2, this value is significantly smaller for the novice group, indicating that there is a need for the novices’ to improve their eye-hand coordination skills, while further supporting an earlier study which reported that novices control on the environment and monitor is lower compared to the experts (54). In this earlier study, the researchers analyzed eye movements of experts and novices and reported that novices concentrate on the surgical display and, as such, lose track of patient’s status, whereas experts also observe these conditions at the same time (54).

Similarly, the results obtained based on the questionnaire data considering the observers’ evaluations on the skill levels of the intermediates in terms of their 3D depth perception and eye-hand coordination are reported as higher than that of the novices.

### Conclusion

This study has two major contributions. Firstly, new hand movement metrics are proposed by adapting an open-source eye-movement classification algorithm (BIT) to the data collected through a computer-based simulation software using a haptic interface and eye-tracker. Secondly, it can be stated that such metrics and eye-hand correlation analyses can be used for the objective assessment of the skill levels required for endoscopic surgery trainees. As studies on eye-hand coordination in the literature are very limited, we believe that analyzing surgeons' hand and eye data jointly is an important contribution to objective evaluations and assessment of their skills and development of some standards for surgical education programs.

In the literature, dispersion-based algorithms are recommended for eye movement event classification while using a low-frequency eye tracking device. However, in this study, an adaptive algorithm is used because the velocity of the eye and hand movements can be different. Due to the ability of automatically defining task- and individual-specific thresholds and being machine and sampling frequency independent, we believe that the BIT algorithm proposed by (38) is suitable for skill-based studies.

### Limitations and Future Work

As it is commonly the case, the number of surgeons in the neurosurgery and ENT departments is very limited, for which reason this study was conducted with 15 participants and only intermediate and novice surgeons. In the future attempts, it may be possible to validate the results of this study with a larger number of participants and also upon a wider range of skill levels. Additionally, in the future, experimental studies should be conducted with the help of scenarios in a larger scope aiming at different tasks with different difficulty levels and under different conditions. For instance, in the present work only both-handed task performances were evaluated; whereas later, the participants’ performances may also be compared in dominant, non-dominant and both hands settings.

As we were unable to obtain the values for the exact time stamps of the provided events, in this study the average values for each metric could be analyzed because of the values provided by the BIT algorithm. Due to this drawback, further analysis needs to be carried out to examine whether the eye and hand events occur in a synchronized way using other appropriate algorithms. Also, the differences among the results of different algorithms can also be further analyzed.

Lastly, Scenario-1 in this study was designed in a more general sense and to gain endoscopic skills, while Scenario-2 was arranged as more specific to the endo-neurosurgery tasks. Accordingly, the experimental study is conducted in the same order as the scenarios (Scenario-1 is performed first and followed by Scenario-2). However, to eliminate the order effect in learning, with more scenarios this effect can be brought under control.

### Ethics and Conflict of Interest

The authors declare that the contents of the article are in agreement with the ethics described in http://biblio.unibe.ch/portale/elibrary/BOP/jemr/ethics.html and that there is no conflict of interest regarding the publication of this paper.

### Acknowledgements

This study was conducted to improve the scenario designs applied in educational materials developed for the endo-neurosurgery education project (ECE: Tubitak 1001, Project No: 112K287). The authors would like to thank the support of TÜBİTAK 1001 program for realizing this research. Our thanks also go to the ECE project team and the Hacettepe University Medical School for their valuable support throughout the research. We also wish to express our sincere thanks to Payam Danesh for his valuable comments on an earlier version of this manuscript.

## References

[b37] Andersson, R., Larsson, L., Holmqvist, K., Stridh, M., & Nyström, M. (2017). One algorithm to rule them all? An evaluation and discussion of ten eye movement event-detection algorithms. Behavior Research Methods, 49(2), 616–637. 10.3758/s13428-016-0738-9 1554-351X 27193160

[b47] Andreatta, P. B., Woodrum, D. T., Gauger, P. G., & Minter, R. M. (2008). LapMentor metrics possess limited construct validity. Simulation in Healthcare, 3(1), 16–25. 10.1097/SIH.0b013e31816366b9 1559-2332 19088638

[b36] Ayodeji, I. D., Schijven, M., Jakimowicz, J., & Greve, J. W. (2007). Face validation of the Simbionix LAP Mentor virtual reality training module and its applicability in the surgical curriculum. Surgical Endoscopy, 21(9), 1641–1649. 10.1007/s00464-007-9219-7 0930-2794 17356944

[b40] Basdogan, C., De, S., Kim, J., Muniyandi, M., Kim, H., & Srinivasan, M. A. (2004). Haptics in minimally invasive surgical simulation and training. IEEE Computer Graphics and Applications, 24(2), 56–64. 10.1109/MCG.2004.1274062 0272-1716 15387229

[b7] Batmaz, A. U., de Mathelin, M., & Dresp-Langley, B. (2017). Seeing virtual while acting real: Visual display and strategy effects on the time and precision of eye-hand coordination. PLoS One, 12(8), e0183789. 10.1371/journal.pone.0183789 1932-6203 28859092 PMC5578485

[b10] Berkenstadt, H., Ziv, A., Barsuk, D., Levine, I., Cohen, A., & Vardi, A. (2003). The use of advanced simulation in the training of anesthesiologists to treat chemical warfare casualties. Anesthesia and Analgesia, 96(6), 1739–1742. 10.1213/01.ANE.0000057027.52664.0B 0003-2999 12761005

[b25] Bishop, D., Kuhn, G., & Maton, C. (2014). Telling people where to look in a soccer-based decision task: A nomothetic approach. Journal of Eye Movement Research, 7(2).1995-8692

[b12] Cagiltay, N. E., Ozcelik, E., Sengul, G., & Berker, M. (2017). Construct and face validity of the educational computer-based environment (ECE) assessment scenarios for basic endoneurosurgery skills. Surgical Endoscopy, 31(11), 4485–4495. 10.1007/s00464-017-5502-4 0930-2794 28389794

[b45] Cohen, J. (1988). Set correlation and contingency tables. Applied Psychological Measurement, 12(4), 425–434. 10.1177/014662168801200410 0146-6216

[b43] Dalmaijer E. Is the low-cost EyeTribe eye tracker any good for research? PeerJ PrePrints, 2014 2167-9843.

[b2] Dankelman, J., Grimbergen, C. K. A., & Stassen, H. G. (2007). New technologies supporting surgical intervenltions and training of surgical skills-a look at projects in europe supporting minimally invasive techniques. IEEE Engineering in Medicine and Biology Magazine, 26(3), 47–52. 10.1109/MEMB.2007.364929 0739-5175 17549920

[b16] Datta, V., Chang, A., Mackay, S., & Darzi, A. (2002). The relationship between motion analysis and surgical technical assessments. American Journal of Surgery, 184(1), 70–73. 10.1016/S0002-9610(02)00891-7 0002-9610 12135725

[b18] D’Angelo, A.-L. D., Rutherford, D. N., Ray, R. D., Laufer, S., Kwan, C., Cohen, E. R., . . . Pugh, C. M. (2015). Idle time: An underdeveloped performance metric for assessing surgical skill. American Journal of Surgery, 209(4), 645–651. 10.1016/j.amjsurg.2014.12.013 0002-9610 25725505 PMC4412306

[b11] Evgeniou, E., & Loizou, P. (2013). Simulation-based surgical education. ANZ Journal of Surgery, 83(9), 619–623. 10.1111/j.1445-2197.2012.06315.x 1445-1433 23088646

[b52] Gegenfurtner, A., Lehtinen, E., & Säljö, R. (2011). Expertise differences in the comprehension of visualizations: A meta-analysis of eye-tracking research in professional domains. Educational Psychology Review, 23(4), 523–552. 10.1007/s10648-011-9174-7 1040-726X

[b9] Gordon, J. A., Wilkerson, W. M., Shaffer, D. W., & Armstrong, E. G. (2001). “Practicing” medicine without risk: Students’ and educators’ responses to high-fidelity patient simulation. Academic Medicine, 76(5), 469–472. 10.1097/00001888-200105000-00019 1040-2446 11346525

[b22] Helsen, W. F., Elliott, D., Starkes, J. L., & Ricker, K. L. (2000). Coupling of eye, finger, elbow, and shoulder movements during manual aiming. Journal of Motor Behavior, 32(3), 241–248. 10.1080/00222890009601375 0022-2895 10975272

[b5] Hermens, F., Flin, R., & Ahmed, I. (2013). Eye movements in surgery: A literature review. Journal of Eye Movement Research, 6(4).1995-8692

[b6] Hernandez, J. D., Bann, S. D., Munz, Y., Moorthy, K., Datta, V., Martin, S., . . . Rockall, T. (2004). Qualitative and quantitative analysis of the learning curve of a simulated surgical task on the da Vinci system. Surgical Endoscopy, 18(3), 372–378. 10.1007/s00464-003-9047-3 0930-2794 14752634

[b23] Jiang, X., Zheng, B., & Atkins, M. S. (2015). Video processing to locate the tooltip position in surgical eye-hand coordination tasks. Surgical Innovation, 22(3), 285–293. 10.1177/1553350614541859 1553-3506 25049318

[b26] Jiang, X., Zheng, B., Bednarik, R., & Atkins, M. S. (2015). Pupil responses to continuous aiming movements. International Journal of Human-Computer Studies, 83, 1–11. 10.1016/j.ijhcs.2015.05.006 1071-5819

[b27] Johnson, B. P., Lum, J. A., Rinehart, N. J., & Fielding, J. (2016). Ocular motor disturbances in autism spectrum disorders: Systematic review and comprehensive meta-analysis. Neuroscience and Biobehavioral Reviews, 69, 260–279. 10.1016/j.neubiorev.2016.08.007 0149-7634 27527824

[b34] Kirk, R. M. (1996). Teaching the craft of operative surgery. Annals of the Royal College of Surgeons of England, 78(1, Suppl), 25–28.0035-8843 8659997

[b46] Laerd Statistics Pearson’s product-moment correlation using SPSS Statistics. Statistical tutorials and software guides. 2017 Available from: https://statistics.laerd.com/

[b1] Lanfranco, A. R., Castellanos, A. E., Desai, J. P., & Meyers, W. C. (2004). Robotic surgery: A current perspective. Annals of Surgery, 239(1), 14–21. 10.1097/01.sla.0000103020.19595.7d 0003-4932 14685095 PMC1356187

[b17] Latko, W. A., Armstrong, T. J., Foulke, J. A., Herrin, G. D., Rabourn, R. A., & Ulin, S. S. (1997). Development and evaluation of an observational method for assessing repetition in hand tasks. American Industrial Hygiene Association Journal, 58(4), 278–285. 10.1080/15428119791012793 0002-8894 9115085

[b53] Law B, Atkins MS, Kirkpatrick AE, Lomax AJ, editors Eye gaze patterns differentiate novice and experts in a virtual laparoscopic surgery training environment. Proceedings of the 2004 symposium on Eye tracking research & applications; 2004: ACM. 10.1145/968363.968370

[b4] Lehmann, K. S., Ritz, J. P., Maass, H., Cakmak, H. K., Kuehnapfel, U. G., Germer, C. T., . . . Buhr, H. J. (2005). A prospective randomized study to test the transfer of basic psychomotor skills from virtual reality to physical reality in a comparable training setting. Annals of Surgery, 241(3), 442–449. 10.1097/01.sla.0000154552.89886.91 0003-4932 15729066 PMC1356982

[b13] Martin, J. A., Regehr, G., Reznick, R., MacRae, H., Murnaghan, J., Hutchison, C., & Brown, M. (1997). Objective structured assessment of technical skill (OSATS) for surgical residents. British Journal of Surgery, 84(2), 273–278.0007-1323 9052454 10.1046/j.1365-2168.1997.02502.x

[b48] McDougall, E. M., Corica, F. A., Boker, J. R., Sala, L. G., Stoliar, G., Borin, J. F., . . . Clayman, R. V. (2006). Construct validity testing of a laparoscopic surgical simulator. Journal of the American College of Surgeons, 202(5), 779–787. 10.1016/j.jamcollsurg.2006.01.004 1072-7515 16648018

[b44] Menekse Dalveren, G. G., & Cagiltay, N. E. (2018). Insights from surgeons’ eye-movement data in a virtual simulation surgical training environment: Effect of experience level and hand conditions. Behaviour & Information Technology, 37(5), 517–537. 10.1080/0144929X.2018.1460399 0144-929X

[b21] Mohamadipanah H, Parthiban C, Law K, Nathwani J, Maulson L, DiMarco S, et al., editors Hand smoothness in laparoscopic surgery correlates to psychomotor skills in virtual reality. Wearable and Implantable Body Sensor Networks (BSN), 2016 IEEE 13th International Conference on; 2016: IEEE. 10.1109/BSN.2016.7516267

[b3] Moore, M. J., & Bennett, C. L. (1995). The learning curve for laparoscopic cholecystectomy. The Southern Surgeons Club. American Journal of Surgery, 170(1), 55–59. 10.1016/S0002-9610(99)80252-9 0002-9610 7793496

[b14] Moorthy, K., Munz, Y., Sarker, S. K., & Darzi, A. (2003). Objective assessment of technical skills in surgery. BMJ (Clinical Research Ed.), 327(7422), 1032–1037. 10.1136/bmj.327.7422.1032 0959-8138 14593041 PMC261663

[b42] Ooms, K., Dupont, L., Lapon, L., & Popelka, S. (2015). Accuracy and precision of fixation locations recorded with the low-cost Eye Tribe tracker in different experimental setups. Journal of Eye Movement Research, 8(1).1995-8692

[b19] Oropesa, I., Sánchez-González, P., Lamata, P., Chmarra, M. K., Pagador, J. B., Sánchez-Margallo, J. A., . . . Gómez, E. J. (2011). Methods and tools for objective assessment of psychomotor skills in laparoscopic surgery. The Journal of Surgical Research, 171(1), e81–e95. 10.1016/j.jss.2011.06.034 0022-4804 21924741

[b20] Oropesa, I., Chmarra, M. K., Sánchez-González, P., Lamata, P., Rodrigues, S. P., Enciso, S., . . . Gómez, E. J. (2013). Relevance of motion-related assessment metrics in laparoscopic surgery. Surgical Innovation, 20(3), 299–312. 10.1177/1553350612459808 1553-3506 22983805

[b33] Oropesa, I., Sánchez-Gonzáez, P., Chmarra, M. K., Lamata, P., Pérez-Rodríguez, R., Jansen, F. W., . . . Gómez, E. J. (2014). Supervised classification of psychomotor competence in minimally invasive surgery based on instruments motion analysis. Surgical Endoscopy, 28(2), 657–670. 10.1007/s00464-013-3226-7 0930-2794 24122243

[b24] Oropesa, I., Sánchez-González, P., Chmarra, M. K., Lamata, P., Fernández, A., Sánchez-Margallo, J. A., . . . Gómez, E. J. (2013). EVA: Laparoscopic instrument tracking based on Endoscopic Video Analysis for psychomotor skills assessment. Surgical Endoscopy, 27(3), 1029–1039. 10.1007/s00464-012-2513-z 0930-2794 23052495

[b28] Parr, J. V. V., Vine, S. J., Harrison, N. R., & Wood, G. (2018). Examining the spatiotemporal disruption to gaze when using a myoelectric prosthetic hand. Journal of Motor Behavior, 50(4), 416–425. 10.1080/00222895.2017.1363703 0022-2895 28925815

[b32] Reiley, C. E., Lin, H. C., Yuh, D. D., & Hager, G. D. (2011). Review of methods for objective surgical skill evaluation. Surgical Endoscopy, 25(2), 356–366. 10.1007/s00464-010-1190-z 0930-2794 20607563

[b29] Schmitt, L. M., Cook, E. H., Sweeney, J. A., & Mosconi, M. W. (2014). Saccadic eye movement abnormalities in autism spectrum disorder indicate dysfunctions in cerebellum and brainstem. Molecular Autism, 5(1), 47. 10.1186/2040-2392-5-47 2040-2392 25400899 PMC4233053

[b39] Silvennoinen, M., Mecklin, J.-P., Saariluoma, P., & Antikainen, T. (2009). Expertise and skill in minimally invasive surgery. Scandinavian Journal of Surgery, 98(4), 209–213. 10.1177/145749690909800403 1457-4969 20218416

[b50] Snyder, L. H., Calton, J. L., Dickinson, A. R., & Lawrence, B. M. (2002). Eye-hand coordination: Saccades are faster when accompanied by a coordinated arm movement. Journal of Neurophysiology, 87(5), 2279–2286. 10.1152/jn.00854.2001 0022-3077 11976367

[b15] Stylopoulos, N., & Vosburgh, K. G. (2007). Assessing technical skill in surgery and endoscopy: A set of metrics and an algorithm (C-PASS) to assess skills in surgical and endoscopic procedures. Surgical Innovation, 14(2), 113–121. 10.1177/1553350607302330 1553-3506 17558017

[b41] The Eye Tribe Basics 2014 Available from: http://theeyetribe.com/dev.theeyetribe.com/dev.theeyetribe.com/general/index.html

[b35] Tien T, Pucher PH, Sodergren MH, Sriskandarajah K, Yang G-Z, Darzi A Eye tracking for skills assessment and training: a systematic review. journal of surgical research. 2014;191(1):169-78. 10.1016/j.jss.2014.04.032 24881471

[b54] Tien G, Atkins MS, Zheng B, Swindells C, editors Measuring situation awareness of surgeons in laparoscopic training. Proceedings of the 2010 symposium on eye-tracking research & applications; 2010: ACM. 10.1145/1743666.1743703

[b51] Uemura M, Tomikawa M, Kumashiro R, Miao T, Souzaki R, Ieiri S, et al. Analysis of hand motion differentiates expert and novice surgeons. journal of surgical research. 2014;188(1):8-13. 10.1016/j.jss.2013.12.009 24418518

[b30] Wang, T.-N., Howe, T.-H., Lin, K.-C., & Hsu, Y.-W. (2014). Hand function and its prognostic factors of very low birth weight preterm children up to a corrected age of 24 months. Research in Developmental Disabilities, 35(2), 322–329. 10.1016/j.ridd.2013.11.023 0891-4222 24316589

[b8] Wentink, B. (2001). Eye-hand coordination in laparoscopy - an overview of experiments and supporting aids. Minimally Invasive Therapy & Allied Technologies, 10(3), 155–162. 10.1080/136457001753192277 1364-5706 16754008

[b31] Wilson, M., McGrath, J., Vine, S., Brewer, J., Defriend, D., & Masters, R. (2010). Psychomotor control in a virtual laparoscopic surgery training environment: Gaze control parameters differentiate novices from experts. Surgical Endoscopy, 24(10), 2458–2464. 10.1007/s00464-010-0986-1 0930-2794 20333405 PMC2945464

[b49] Yamaguchi, S., Konishi, K., Yasunaga, T., Yoshida, D., Kinjo, N., Kobayashi, K., . . . Hashizume, M. (2007). Construct validity for eye-hand coordination skill on a virtual reality laparoscopic surgical simulator. Surgical Endoscopy, 21(12), 2253–2257. 10.1007/s00464-007-9362-1 0930-2794 17479319

[b38] van der Lans, R., Wedel, M., & Pieters, R. (2011). Defining eye-fixation sequences across individuals and tasks: The Binocular-Individual Threshold (BIT) algorithm. Behavior Research Methods, 43(1), 239–257. 10.3758/s13428-010-0031-2 1554-351X 21287116 PMC3048294

